# ATP6AP2 is robustly expressed in pancreatic β cells and neuroendocrine tumors, and plays a role in maintaining cellular viability

**DOI:** 10.1038/s41598-023-36265-3

**Published:** 2023-06-07

**Authors:** Tomomi Taguchi, Kaori Kimura, Agena Suzuki, Rei Fujishima, Naoya Shimizu, Ayako Hoshiyama, Tsuguto Masaki, Mitsuko Inoue, Yukiko Kato, Takebe Satomi, Koji Takano, Tasuku Imada, Shugo Sasaki, Takeshi Miyatsuka

**Affiliations:** 1 Department of Endocrinology, Diabetes and Metabolism, Kitasato University School of Medicine, 1-15-1 Kitazato, Minami-Ku, Sagamihara, Kanagawa 252-0374 Japan; 2 Health Care Center, Kitasato University, 1-15-1 Kitazato, Minami-Ku, Sagamihara, Kanagawa 252-0374 Japan; 3 Department of Metabolic Medicine, Osaka University Graduate School of Medicine, 2-2 Yamadaoka, Suita, Osaka 565-0871 Japan

**Keywords:** Endocrinology, Oncology

## Abstract

ATP6AP2, also known as (pro)renin receptor, has been shown to be expressed in several tissues including pancreatic β cells. Whereas ATP6AP2 plays an important role in regulating insulin secretion in mouse pancreatic β cells, the expression profiles and roles of ATP6AP2 in human pancreatic endocrine cells and neuroendocrine tumor cells remain unclear. Here in this study, we investigated the expression profiles of ATP6AP2 in pancreatic endocrine cells, and found that ATP6AP2 is robustly expressed in pancreatic insulinoma cells as well as in normal β cells. Although ATP6AP2 was also expressed in low-grade neuroendocrine tumors, it was not or faintly detected in intermediate- and high-grade neuroendocrine tumors. Knockdown experiments of the *Atp6ap2* gene in rat insulinoma-derived INS-1 cells demonstrated decreased cell viability accompanied by a significant increase in apoptotic cells. Taken together, these findings suggest that ATP6AP2 plays a role in maintaining cellular homeostasis in insulinoma cells, which could lead to possible therapeutic approaches for endocrine tumors.

## Introduction

ATPase H^+^ transporting accessory protein 2 (Atp6ap2), also known as (pro)renin receptor, was first cloned as a functional renin receptor. (Pro)renin was reported to bind to ATP6AP2 with a higher affinity than renin to activate proliferation functions, such as the MAPK/ERK pathway and the PI3-AKT-mTOR pathway, independently from angiotensin II^1,2^. Subsequent studies demonstrated the additional functions of ATP6AP2 as an accessory protein of H^+^-ATPase (V-ATPase), a component of the Wnt receptor complex, which controls cell polarity via the atypical partitioning defective homologue system. V-ATPase is a major proton pump that controls proton homeostasis in all cell type and in various subcellular compartments^3^. For example, ATP6AP2 in pituitary cells is reported to regulate growth hormone release via V-ATPase^4^. Previous studies have demonstrated that suppression of the *ATP6AP2* gene induces apoptosis in pancreatic cancer^5,6^, and colorectal cancer^7^. In addition, conditional ablation of *Atp6ap2* gene in mice has further demonstrated essential roles of *Atp6ap2* in maintaining cellular homeostasis in many cell types, such as cardiomyocytes^8^, T cells^9^, neurons^10^, and pancreatic β cells^11^.

Neuroendocrine neoplasms (NENs), which includes the well-differentiated family “neuroendocrine tumor (NET)” and poorly differentiated family “neuroendocrine carcinoma (NEC)”, occur almost everywhere in the body and produce various hormones. Insulinoma is the most common type of pancreatic neuroendocrine neoplasms (PanNENs)^12,13^, accounting for about 20% of all PanNENs. Excessive insulin secretion by insulinoma causes hyperinsulinemic hypoglycemia, which leads to neuroglycopenic, autonomic, adrenergic, and cholinergic symptoms. Whereas about 90% of insulinoma patients have benign and solitary tumors, the other 10% have malignant tumors^14^. Surgical resection has remained the only curative treatment for patients with insulinoma, and alternative treatments are medications, such as somatostatin analogues, streptozotocin and everolimus^15–17^, although no medication can fully inhibit tumor progression and prevent subsequent hypoglycemia.

As the suppression of ATP6AP2 has been reported to induce apoptosis in various types of tumors^5,6^, as described above, we hypothesized that a loss-of-function approach for *Atp6ap2* may also inhibit the tumor growth in insulinoma cells. Therefore, we analyzed the expression profiles of ATP6AP2 in pancreatic endocrine cells and insulinoma cells, and aimed to clarify whether the knockdown approach for *Atp6ap2* can reduce the viability of insulinoma cells, which may lead to the development of novel therapeutic approaches.

## Results

### ATP6AP2 proteins are expressed in human pancreatic endocrine cells

**Figure 1:**
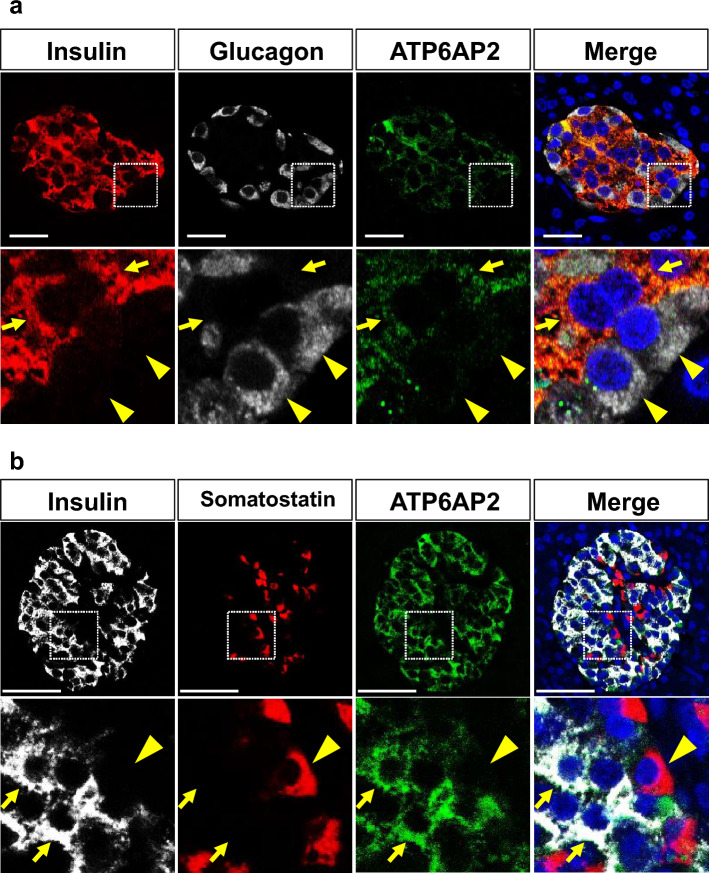
A normal islet stained for ATP6AP2, insulin, and glucagon. (**a**) Immunostaining for ATP6AP2 (green), insulin (red), and glucagon (white) in a normal islet near the tumor lesion of human insulinoma. Nuclei are labeled with DAPI (blue). The arrowheads indicate glucagon-positive, ATP6AP2-negative cells, and the arrows indicates insulin/ATP6AP2 double-positive cells. (**b**) Immunostaining for ATP6AP2 (green), insulin (white), and somatostatin (red) in a normal islet near the tumor lesion of human insulinoma. Nuclei are labeled with DAPI (blue). The arrowhead indicates somatostatin-positive, ATP6AP2-negative cells, and the arrows indicates insulin/ATP6AP2 double-positive cells. Magnified images of the dotted square regions are shown below each image. Scale bars, 50 μm.

It has been reported that V-ATPase and its accessory protein ATP6AP2 are expressed in mouse islets^11,18^. In addition, single-cell RNA sequencing of human pancreata from five healthy subjects demonstrated that *ATP6AP2* mRNAs are highly expressed in various types of endocrine cells in the pancreas^19^. To further confirm that the ATP6AP2 protein is indeed expressed in human endocrine cells, including tumor cells, immunohistological analysis was performed in human pancreata from patients with insulinoma and nonfunctioning NET. The expression of ATP6AP2 proteins was clearly detected in insulin-expressing cells of normal islets near the insulinoma lesions (Fig. 1a,b) and nonfunctioning NETs (Fig. S1), whereas it was not or faintly detected in glucagon-expressing cells and somatostatin-expressing cells in the same regions. In addition, immunostaining in rat insulinoma cell line INS-1 cells demonstrated ATP6AP2 expression in the cytosol of INS-1 cells (Fig. S2).

### ATP6AP2 proteins are expressed in insulinomas and nonfunctioning NETs

**Figure 2:**
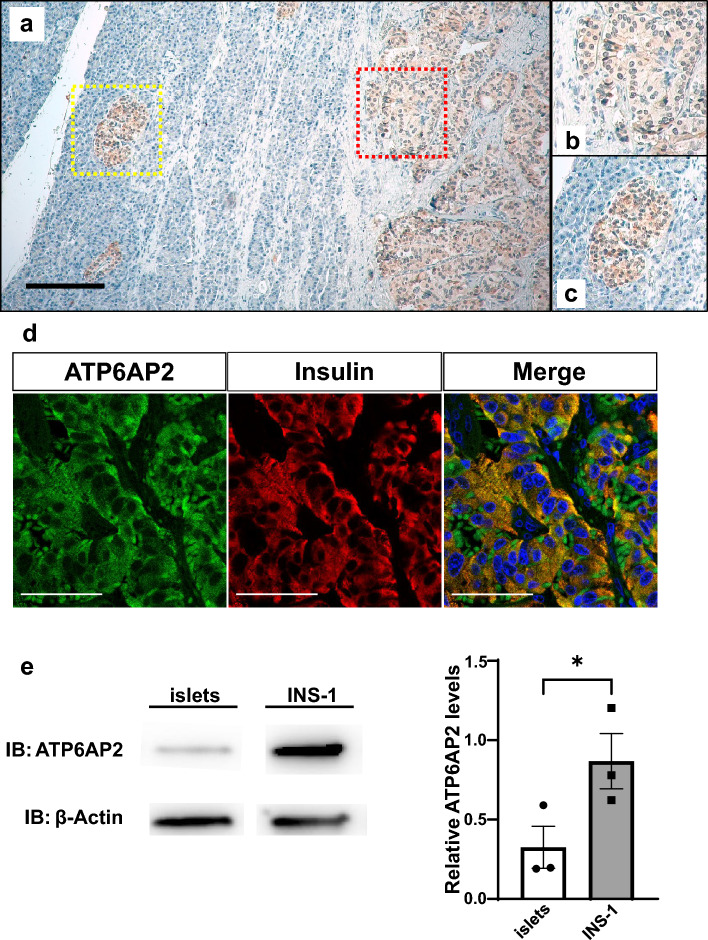
Insulinoma cells stained for ATP6AP2. (**a**) A human pancreatic section containing both normal islets and insulinoma lesions was stained for ATP6AP2. Magnified images of the red- and yellow-dotted square regions are shown in (**b**,**c**), respectively. Scale bars, 200 μm. Nuclei were labeled with hematoxylin (blue). (**d**) Immunostaining for ATP6AP2 (green) and insulin (red) in an insulinoma lesion. Nuclei were labeled with DAPI (blue). Scale bars, 50 μm. (**e**) ATP6AP2 proteins in rat INS-1 and mouse islets were quantified. *p < 0.05, n = 3 in each group.

To further investigate whether ATP6AP2 proteins are expressed in endocrine tumor cells in the pancreata, immunohistological analysis was performed in human pancreata from 10 patients with insulinoma and 31 patients with nonfunctioning NET. The clinical characteristics of the patients are listed in Table 1 and the Supplementary Tables. Immunostaining against ATP6AP2 demonstrated the robust expression of ATP6AP2 in insulinoma cells as well as in non-tumor islet cells of the same section, whereas non-tumor cells surrounding the insulinoma cells were not stained with ATP6AP2 (Fig. 2a–c). Coimmunostaining against ATP6AP2 and insulin demonstrated that all ATP6AP2-expressing cells overlapped with insulin (Fig. 2d). These findings suggest that ATP6AP2 proteins are highly and specifically expressed in insulin-expressing cells in pancreatic sections of insulinoma. Furthermore, immunoblotting against ATP6AP2 demonstrated a significantly higher level of ATP6AP2 protein expression in INS-1 cells compared with in islets isolated from C57BL/6 J mice (Fig. 2e, Fig. S3).

**Table 1:** Clinical characteristics of patients with pancreatic tumors.

	Insulinoma	Nonfunctioning NET
n	10	31
Age (years)	59.4 ± 22.2	58.7 ± 10.5
Men (%)	30.0	64.5
Size (mm)	16 ± 0.44	19 ± 1.44
Grade		
G1 (Ki67 index < 3)	9	21
G2 (3 ≤ Ki67 index < 20)	1	9
G3 (Ki67 index ≥ 20)	0	1
Mean Ki67 index (%)	1.5 ± 0.71	4.36 ± 9.03

Pathological diagnosis of pancreatic neuroendocrine neoplasms was based on the WHO 2019 classification. Data are mean ± SD.

**Figure 3:**
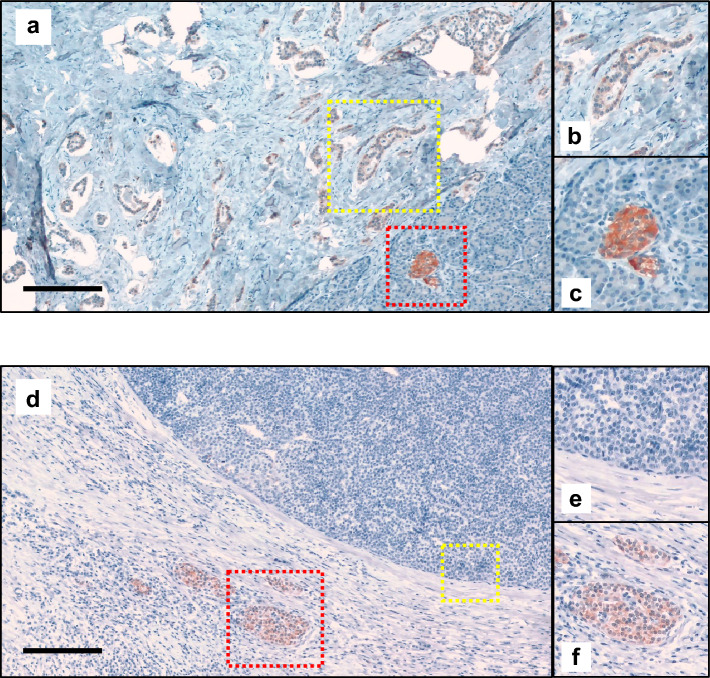
Nonfunctioning NETs stained for ATP6AP2. Pancreatic sections from human nonfunctioning neuroendocrine tumors, classified as NET G1 (**a**–**c**) or NET G3 (**d**–**f**), were stained for ATP6AP2. The yellow-square regions including tumor cells are magnified in (**b**,**e**), and red-square regions including normal islet cells are magnified in (**c**,**f**). Scale bars, 200 μm. (**g**) Immunostaining for ATP6AP2 (green) and insulin (red) in NET lesions. Nuclei were labeled with DAPI (blue). Scale bars, 200 μm.

We further performed immunostaining against ATP6AP2 in pancreatic sections from patients with nonfunctioning NET. Whereas low-grade (G1) NET cells were clearly stained for ATP6AP2 (Fig. 3a–c), G2/G3 NET cells in which Ki67 index was greater than 10%, were hardly stained for ATP6AP2 (Fig. 3d–f, Table 2, Fig. S4). Immunostaining for ATP6AP2 and insulin in different types of NET demonstrated that ATP6AP2 proteins were clearly detected in normal islets and tumor cells in G1 NET, but not in G2/G3 NET cells (Fig. S3), which suggests that the expression level of ATP6AP2 is likely to be correlated with proliferation capacity of NET cells, and can be a therapeutic target in certain types of NETs.

**Table 2:** Number of patients with ATP6AP2-positive PanNET cells (%).

	Insulinoma	Nonfunctioning NET
Grade		
G1 (Ki67 < 3)	9 out of 9 (100)	18 out of 21 (85.7)
G2		
3 ≤ Ki67 index < 10	1 out of 1 (100)	7 out of 7 (100)
10 ≤ Ki67 index < 20	N/A	0 out of 2 (0)
G3 (Ki67 index ≥ 20)	N/A	0 out of 1 (0)
IRS		
Intensity	1.78 ± 0.67	1.32 ± 0.87
Proportion	3.33 ± 0.71	2.26 ± 1.48
Total score	6 ± 2.5	3.77 ± 3.28

Pathological diagnosis of pancreatic neuroendocrine neoplasms was based on the WHO 2019 classification.

*N/A* not available, *NET* neuroendocrine tumor, *IRS* immunoreactivity score.

### Suppression of *ATP6AP2* gene expression induces the apoptosis of INS-1 cells

**Figure 4:**
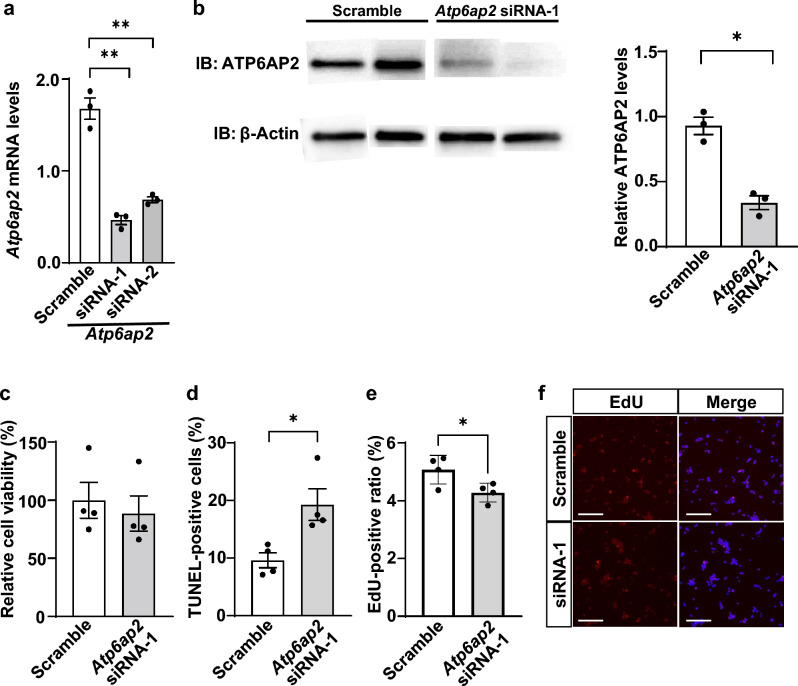
Effect of *Atp6ap2* siRNA transfection on cell viability and apoptosis in rat insulinoma cells. Rat insulinoma cells (INS-1) were transfected with scramble control siRNA (Scramble), *Atp6ap2* siRNA-1, and *Atp6ap2* siRNA-2 for 48 h, and *Atp6ap2* mRNAs (**a**) and ATP6AP2 proteins (**b**) were quantified. **p < 0.01, n = 3 in each group. (**c**) Relative cell viability of INS-1 cells transfected with *Atp6ap2* siRNA-1 was evaluated by the WST-1 assay, and normalized to that of control INS-1 cells transfected with scramble siRNA. (**d**,**e**) The percentages of TUNEL-positive cells (**d**) and EdU-positive cells (**e**) were quantified with INS-1 cells seeded onto 12-well plates at a density of 5.0 × 10^5^ /well. *p < 0.05; n = 4 in each group. (**f**) Representative images of INS-1 cells treated with *Atp6ap2* siRNA-1 or scramble. Scale bars, 200 μm. All data are shown as the mean ± SE.

To investigate the role of ATP6AP2 in insulinoma cells, *ATP6AP2* mRNAs were knocked down in the rat insulinoma cell line INS-1 using small interfering RNAs (siRNAs). As shown in Fig. 4a, both *Atp6ap2* siRNA-1 and siRNA-2 significantly suppressed the expression of *Atp6ap2* mRNAs compared with scrambled siRNAs. In addition, immunoblot analysis resulted in a significant decrease in ATP6AP2 proteins by *Atp6ap2* siRNA-1 (Fig. 4b, Supplementary Fig. 3). When cell viability of INS-1 cells was evaluated by the WST-1 assay, there was no significant difference in cell viability between cells treated with *Atp6ap2* siRNA-1 and those treated with scrambled siRNAs (Fig. 4c), whereas terminal deoxynucleotidyl transferase dUTP nick-end labeling (TUNEL) staining demonstrated that the number of TUNEL-positive cells was significantly increased by *Atp6ap2* siRNA-1 treatment (Fig. 4d). In addition, 5-ethynyl-2-deoxyuridine (EdU) staining showed that the number of EdU-positive cells was significantly decreased in the cells treated with *Atp6ap2* siRNA-1 (Fig. 4e,f). Taken together, these results suggested that *ATP6AP2* likely plays a role in maintaining the cellular viability of insulinoma cells.

### Suppression of *Atp6ap2* gene expression and a somatostatin analogue nonadditively induced apoptosis

**Figure 5:**
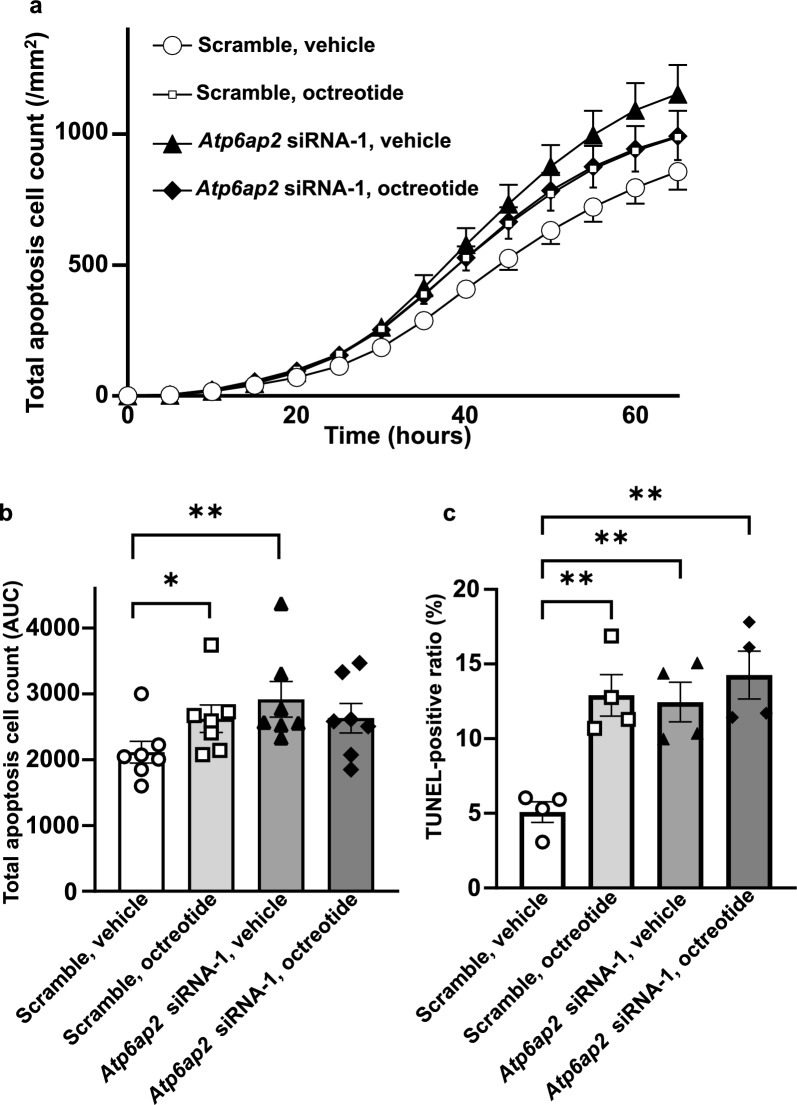
Effects of *Atp6ap2* siRNA transfection and/or octreotide on apoptosis in rat insulinoma cells. (**a**) Real-time quantification of cellular apoptosis using IncuCyte ZOOM in INS-1 cells transfected with *Atp6ap2* siRNAs or scramble siRNAs, together with octreotide or vehicle. (**b**) Area under the curve (AUC) of the apoptotic cell number between 0 and 65 h hours was calculated using Turkey’s multiple comparisons test. *p < 0.05, **p < 0.01; n = 7 in each group. (**c**) INS-1 cells were seeded onto 12-well plates at a density of 5.0 × 10^5^/well, and then treated with control siRNA (scramble) or *Atp6ap2* siRNA-1, together with octreotide or vehicle, for 72 h. The percentages of TUNEL-positive cells were then quantified. **p < 0.01; n = 4 in each group. All data are shown as the mean ± SE.

To further investigate whether the suppression of *Atp6ap2* gene expression shows any synergistic effects in combination with already established anti-tumor treatments, INS-1 cells were treated with *Atp6ap2* siRNA-1 and/or octreotide, a somatostatin analogue, and the number of apoptotic cells was sequentially quantified with IncuCyte®, a high‑throughput device that can acquire and view culture cell images over time. Whereas both treatment with *Atp6ap2* siRNA-1 and octreotide significantly increased the number of apoptotic cells compared with treatment with scrambled siRNAs (Fig. 5a,b), which was confirmed by TUNEL staining (Fig. 5c), there were no additive effects of *Atp6ap2* siRNA-1 and octreotide treatment on cellular apoptosis.

## Discussion

While ATP6AP2 has been reported to play a role in regulating the viability of various types of tumor cells, such as pancreatic cancer^5^ and glioblastoma^6^, the expression profiles of ATP6AP2, and the potential role of ATP6AP2 in pancreatic endocrine cells, including tumor cells, remains unclear. In this study, histological analysis demonstrated the robust expression of ATP6AP2 in human β cells, which is consistent with previous findings in mouse and human pancreata^11,18,20^. Whereas single-cell RNA sequencing resulted in the widespread expression of *ATP6AP2* mRNAs in pancreatic endocrine cells as well as acinar cells^19^, we could hardly detect the expression levels of ATP6AP2 proteins in α cells, δ cells, and acinar cells in our present study (Fig. 1). Therefore, it is possible that the post-transcriptional modification of *ATP6AP2* differs between β cells and non-β-cells in the pancreas. On the other hand, another previous study reported that the ATP6AP2 protein was detected in a proportion of α cells as well as in β cells in human islets^20^, which implies that ATP6AP2 can be expressed in some α cells under certain conditions.

ATP6AP2 was found to be expressed not only in pancreatic β cells but also in insulinoma cells in all patients (Fig. 2, Table 2). On the other hand, in some patients with nonfunctioning NETs, in which the Ki67 index was greater than 10%, there were few cells stained for ATP6AP2 (Fig. 3d–f, Table 2), showing that the expression of ATP6AP2 was negatively correlated with tumor growth in nonfunctioning NETs. This is in contrast with previous studies, which showed that ATP6AP2 expression was positively correlated with the Ki67-labeling index^6^, and that systemic administration of anti-ATP6AP2 antibodies into mice bearing subcutaneous pancreatic adenocarcinoma cells significantly decreased the number of Ki67-positive cells^21^. In addition, EdU-staining demonstrated that the suppression of *Atp6ap2* gene expression inhibited a proliferation of INS-1 cells (Fig. 4e), which is consistent with previous reports^6,21^. Whereas *Atp6ap2* knockdown resulted in no significant effects in the WST-1 assay (Fig. 4c), it significantly increased the number of TUNEL-positive cells (Fig. 4d). As the WST-1 assay reflects metabolic activity as well as cell viability, compensative metabolic processes may affect the results of the WST-1 assay. Further metabolomics analysis would provide better insight into the molecular mechanisms underlying these results. As it has been reported that *Atp6ap2* deletion does not induce cellular apoptosis in primary β cells^11^, it is possible that the role of ATP6AP2 in the cell viability may be different between primary β cells and insulinoma cells. Furthermore, the expression levels of *Atp6ap2* mRNAs was shown to be positively associated with the progression of pancreatic ductal adenocarcinoma^22^. These results imply that the role of ATP6AP2 in cellular proliferation may depend on the cell types and features of tumor cells. These findings, together with in vitro data that the suppression of *Atp6ap2* gene expression induces apoptosis in INS-1 insulinoma cells (Figs. 4 and 5), suggest that the *Atp6ap2* gene may be a possible therapeutic target for insulinoma and early stage NETs, but not for advanced NETs with higher proliferation activity.

The downregulation of *ATP6AP2* resulted in increased cellular apoptosis in insulinoma cells (Fig. 5), which is consistent with previous findings in other cell types, such as pancreatic ductal adenocarcinoma (PDAC) cells^5^, bronchial epithelial cells^23^, and nephron cells^24^. Silencing of *ATP6AP2* mRNAs in PDAC cells and bronchial epithelial cells attenuated activation of the Wnt/β-catenin signaling pathway, and increased apoptosis^5,23^. Further studies will be needed to clarify the possible association between ATP6AP2 and the Wnt/β-catenin signaling pathway in pancreatic β cells and NET cells.

As ATP6AP2 is highly expressed in many cell types and regulates their viability, it is possible that the suppression of ATP6AP2 will have critical effects on cell viability, similarly to cytotoxic anticancer agents, such as cisplatin, gemcitabine, irinotecan and paclitaxel, having many adverse effects. On the other hand, such cytotoxic agents have been shown to have beneficial effects on killing cancer cells, beyond their adverse effects. As tumor cells in G1 NETs express high levels of ATP6AP2, optimal levels of ATP6AP2 suppression are expected to target NET cells without having any critical adverse effects in other cell types.

*Atp6ap2* siRNAs induced the cellular apoptosis in insulinoma cells as potently as the somatostatin analogue octreotide, although there were no additive effects between *Atp6ap2* siRNAs and octreotide (Fig. 5). Somatostatin analogues, such as octreotide and lanreotide, can be used as the first-line therapy for malignant insulinomas due to their antiproliferative effect^25^. While somatostatin analogues (SSAs) are able to control symptoms in 35–50% of insulinoma patients, their efficacy is limited partly because of tachyphylaxis^26^. Furthermore, other medications, such as streptozotocin and everolimus, cannot completely suppress tumor growth and subsequent hypoglycemia in patients with insulinoma. Therefore, it is expected that targeting the *Atp6ap2* gene would be an additional option for patients with insulinoma who cannot be cured by current therapies. Other synergistic interactions between *Atp6ap2* suppression and various medications should be investigated in the future for the development of more effective strategies to cure insulinoma and pancreatic NETs.

## Materials and methods

### Clinical data and tissue specimens

Specimens of insulinoma and nonfunctioning NET were obtained from 41 Japanese patients, who underwent surgical resection at Kitasato University Hospital between 2006 and 2020. 10 patients with insulinoma were diagnosed based on typical clinical features during hypoglycemia and high insulin levels. A histological diagnosis of insulinoma and nonfunctioning NETs was based on hematoxylin and eosin staining, and immunostaining. The study protocol and a waiver of written informed consent were approved by Clinical Research Review Board of The Kitasato Institute (study approval no.: B20-377). Patients’ data was obtained through an opt-out methodology. All study methods were performed in accordance with the relevant guidelines and regulations of this organization as well as the Ethical Guidelines for Medical and Health Research Involving Human Subjects in Japan.

### Immunohistochemical analysis

Tissue sections were deparaffinized and microwaved at 95 °C for 20 min in citrate buffer (pH 6.0). Slides were blocked using Endogenous Avidin/Biotin Blocking Kit (Nichirei Bioscience, Tokyo, Japan) and 1% horse serum. The primary antibodies used in this study were the following: guinea pig anti-insulin (1:5; Carpinteria, CA, USA), mouse anti-glucagon (1:1,000; Sigma-Aldrich, Munich, Germany), and rabbit anti-ATP6AP2 (1:75; Sigma-Aldrich). Preliminary immunostaining against ATP6AP2 alone, and against both insulin and ATP6AP2, was performed using serial sections, to confirm that the staining features were the same between the two protocols. The secondary antibodies used in this study were Alexa Fluor 488-conjugated anti-rabbit IgG (1:200, Thermo Fisher Scientific, Waltham, MA, USA), Alexa Fluor 546-conjugated anti-guinea pig IgG (1:200, Thermo Fisher Scientific), and Alexa Fluor 647-conjugated anti-mouse IgG (1:200, Thermo Fisher Scientific). Images were acquired using an LSM710 confocal microscope (Carl Zeiss, Jena, Germany).

The insulinoma and nonfunctioning NETs were scored semiquantitatively using a well-established immunoreactivity scoring system (IRS). The IRS is calculated by multiplying the percentage of positive cells (4, > 80%; 3, 51–80%; 2, 10–50%; 1, 0–10%; 0, 0%), and the intensity of the staining (3, strong; 2, moderate; 1, mild; and 0, no staining), which results in IRS scores between 0 (no staining) and 12 (maximum staining).

### In vitro experiments with INS-1 cells

The rat insulinoma cell line INS-1 was a generous gift from Professor C. B. Wollheim (University Medical Center, Geneva, Switzerland). INS-1 cells were cultured in RPMI-1640 medium supplemented with 10% fetal bovine serum, 1 mM sodium pyruvate, 10 mM HEPES, 50 U/mL penicillin/streptomycin, and 50 μM 2- mercaptoethanol in a humidified atmosphere containing 5% CO_2_ at 37 °C. After the cell density reached 80% confluence, the cells were passaged once every seven days.

For knockdown experiments using *Atp6ap2* siRNAs, INS-1 cells were seeded onto 6-well plates at a density of 1 × 10^6^ /well. Lipofectamine RNAi max (Thermo Fisher Scientific) was used for siRNAs transfection according to the manufacturer’s instructions. For the assessment of cell viability, INS-1 cells were seeded onto 96-well plates at a density of 5.0 × 10^4^/well, and then analyzed using the WST-1 Cell Proliferation Assay Kit (Takara Bio, Shiga, Japan).

### Real-time PCR

Total RNA was isolated from INS-1 cells using TRIzol reagent (Invitrogen, Carlsbad, CA, USA), reverse transcribed with a first-strand cDNA synthesis kit (Takara Bio), and quantified using CFX96 Touch™ Real-Time PCR Detection System (Bio-Rad Laboratories, Hercules, CA, USA). Expression levels of *Atp6ap2* mRNAs were normalized to the expression levels of β-glucuronidase (*Gusb*).

### Immunoblot analysis

Mouse islets were isolated from adult C57BL/6J mice at the age of 4 to 6 months. INS-1 cells were harvested 48 h after transfection of *Atp6ap2* siRNAs, and immunoblot analysis was performed. The primary antibodies used in this experiment were the following: rabbit anti-ATP6AP2 antibody (1:1,000, Sigma-Aldrich) and an anti-β-actin antibody (1:3,000, Abcam, Cambridge, UK). Intensities of the bands in immunoblots were measured by densitometry analysis using Image J software.

### TUNEL staining

INS-1 cells transfected with *Atp6ap2* siRNAs were cultured for 48 h and the TUNEL assay was performed using ApopTag Fluorescein Direct In Situ Apoptosis Detection Kit (EMD Millipore, Burlington, Massachusetts, USA) according to the manufacturer’s instructions. TUNEL-positive cells were imaged by laser-scanning confocal microscopy using an LSM710 confocal microscope (Carl Zeiss).

### EdU cell proliferation assay

To assess the number of proliferating cells, INS-1 cells were seeded onto 12-well plates at a density of 5.0 × 10^5^/well, incubated with EdU for two hours, and then fixed with formaldehyde. The EdU-labeled cells were detected using the Click-iT EdU Cell Proliferation Kit for Imaging (Thermo Fisher Scientific) according to the manufacturer’s instructions.

### Real-time imaging of cell death

INS-1 cells (5 × 10^4^ cells/well) were plated onto 96-well plates, and treated with *Atp6ap2* siRNAs or octreotide. The treatment reagents containing the Incucyte® Caspase 3/7 reagent, diluted in cell culture media (1:1,000 dilution), were added to each well, and the number of apoptotic cells was continuously monitored using a live cell analysis system (IncuCyte ZOOM; Essen Bioscience, Ann Arbor, MI, USA).

### Statistical analysis

Statistical analyses were performed using GraphPad Prism 9.2 software. Comparisons of two samples were performed by the unpaired two-tailed *t*-test. Multiple groups were analyzed by one-way ANOVA with a multiple comparison test, followed by the Tukey–Kramer’s post-hoc test. A *p*-value of less than 0.05 was considered to indicate a statistically significant difference between two groups.

## Supplementary Information

Supplementary Information.

## Data Availability

The datasets used and/or analysed during the current study available from the corresponding author on reasonable request.
