# Supplementary material for: ATP6AP2 is robustly expressed in pancreatic β cells and neuroendocrine tumors, and plays a role in maintaining cellular viability
Source: Sci Rep. 2023 Jun 7;13:9260. doi: 10.1038/s41598-023-36265-3 (PMC10247715; doi:10.1038/s41598-023-36265-3)
Supplement: Supplementary file 1 — Supplementary Information. [file 41598_2023_36265_MOESM1_ESM.pdf]

Figure S1

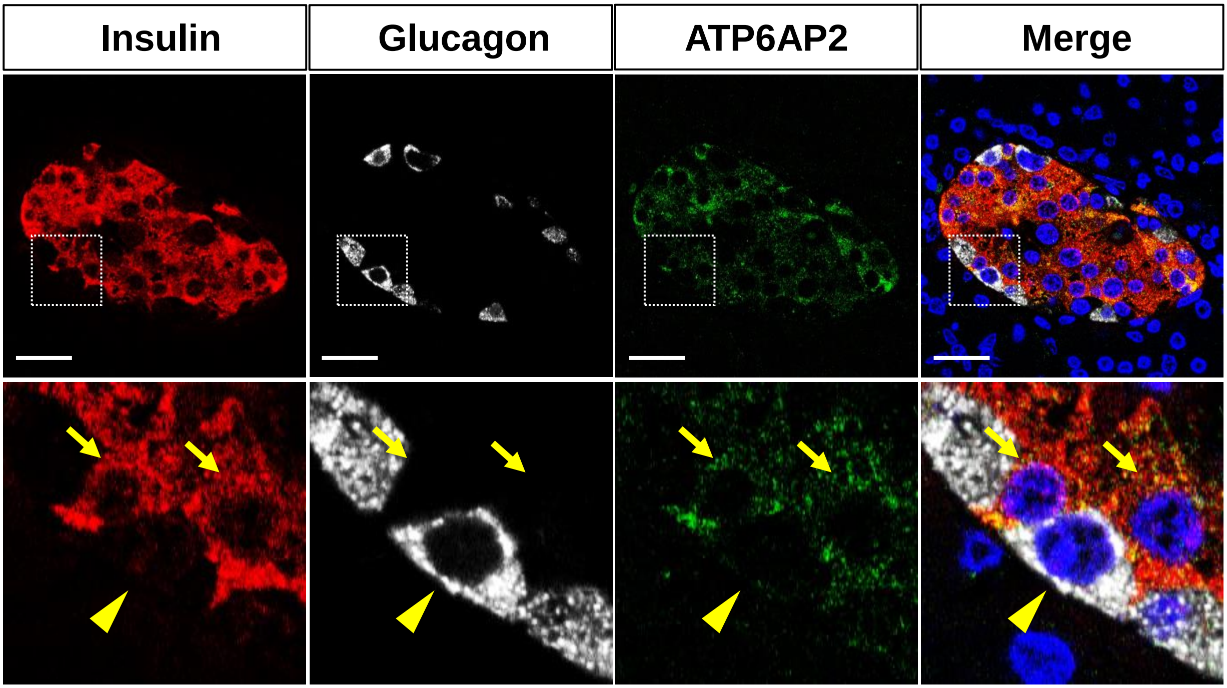

Figure S1. A normal islet around nonfunctioning NET stained for ATP6AP2, insulin, and glucagon. Immunostaining for ATP6AP2 (green), insulin (red), and glucagon (white) in a normal islet near the tumor lesion of human nonfunctioning NET. Nuclei are labeled with DAPI (blue). The arrowhead indicates glucagon-positive, ATP6AP2-negative cells, and arrows indicate insulin/ATP6AP2 double-positive cells. Magnified images of the dotted square regions are shown below each image. Scale bars, 50 μm.

Figure S2

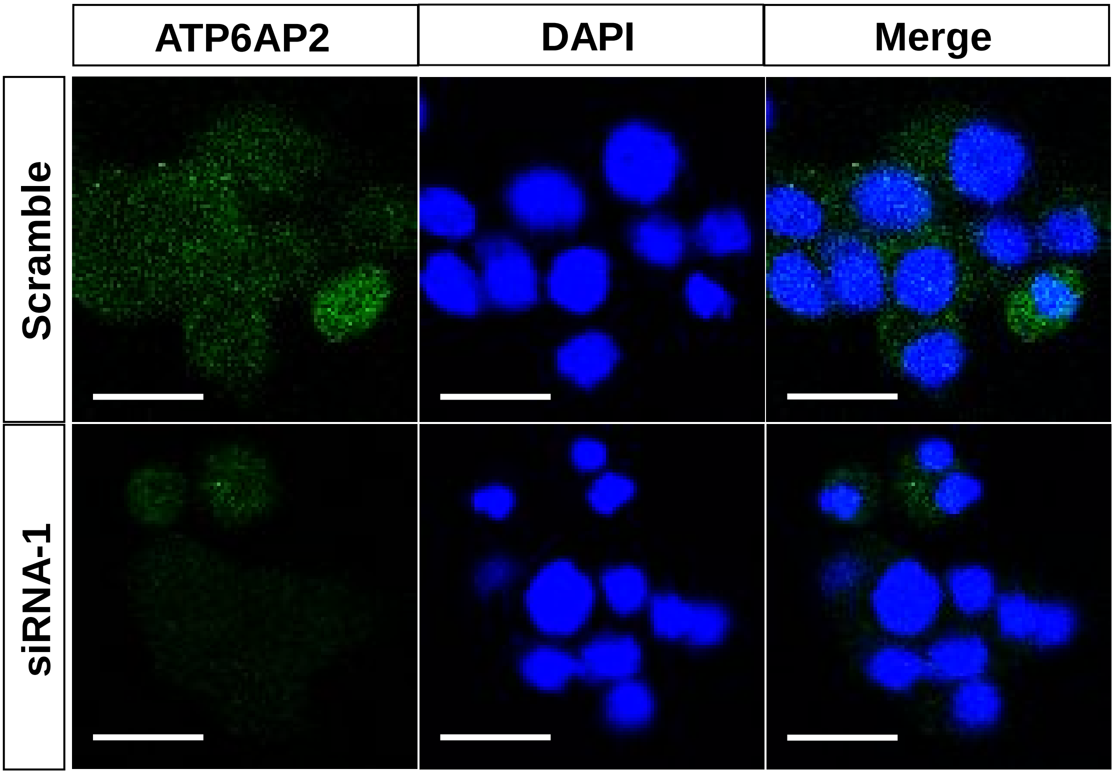

Figure S2. INS-1 cells stained for ATP6AP2.
Representative images of INS-1 cells treated with Atp6ap2 siRNA-1 or scramble, which were immunostained for ATP6AP2 (green). Nuclei are labeled with DAPI (blue). Scale bars, 20 μm.

Figure S3

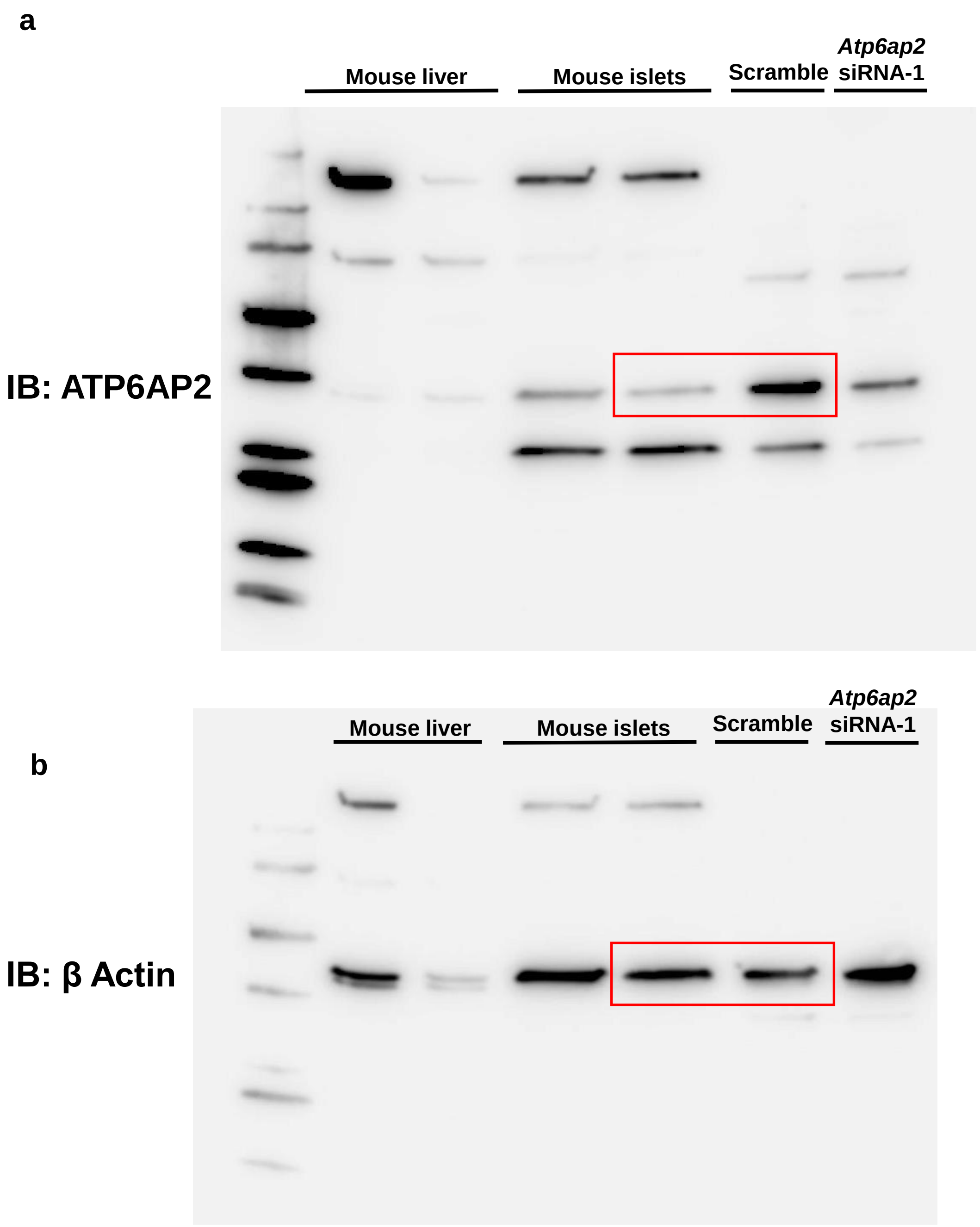

Figure S3. Uncropped images of the immunoblotting shown in Figure 2e.
Red boxes indicate the cropped portion of each immunoblot presented in Figure 2e.

Figure S4

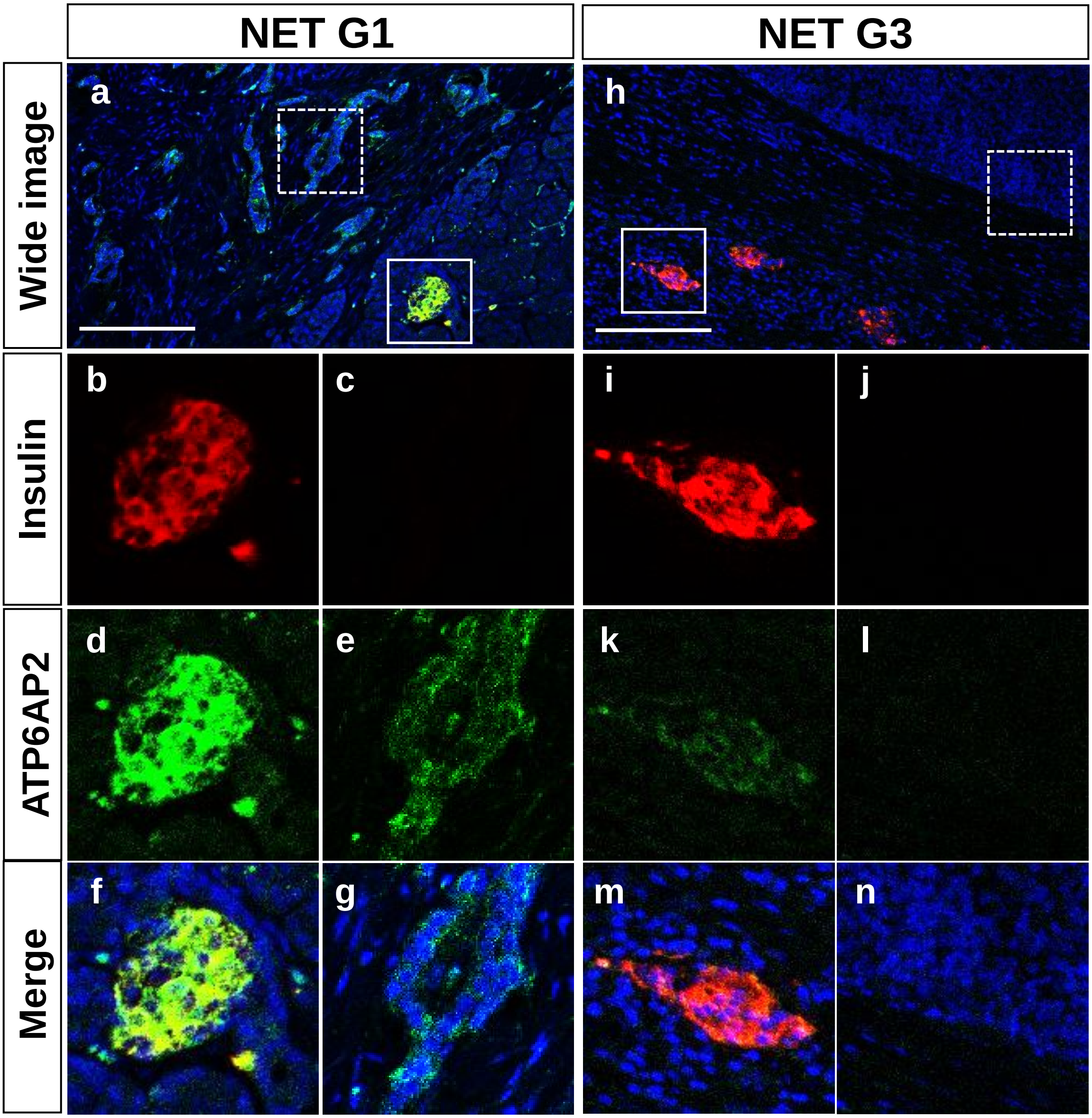

Figure S4. Nonfunctioning NETs stained for ATP6AP2 and insulin.
Pancreatic sections from human NETs, classified as NET G1 (a-g) or NET G3 (h-n), were stained for ATP6AP2 and insulin. The solid-square regions including normal islet cells are magnified in (b), (d), (f), (i), (k) and (m). The dotted-square regions including tumor cells are magnified in (c), (e), (g), (j), (l), and (n). Nuclei were labeled with DAPI (blue). Scale bars, 200 μm.

Figure S5

a

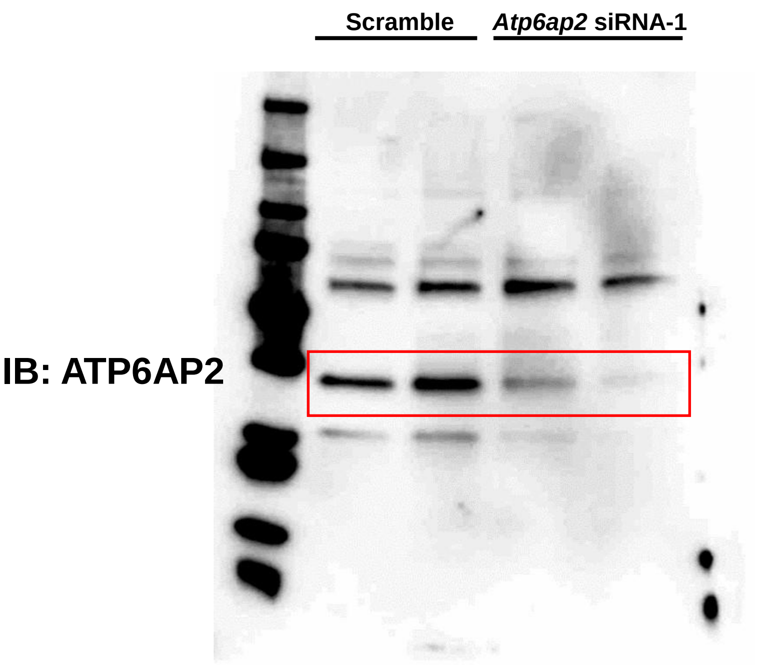

b

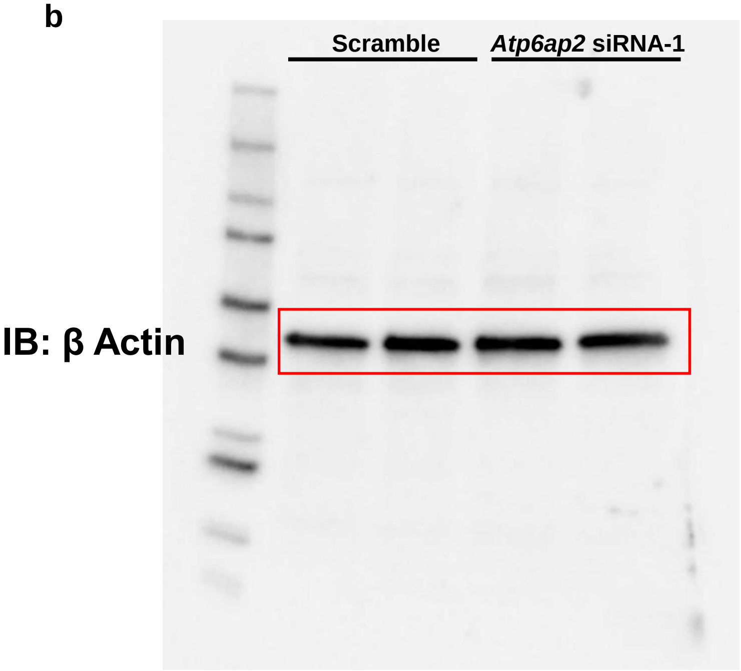

Figure S5. Uncropped images of the immunoblotting shown in Figure 4b.
Red boxes indicate the cropped portion of each immunoblot presented in Figure 4b.

Supplementary table 1

The staging was based on the American Joint Committee on Cancer (AJCC) eithth edition. pT, pathological primary tumor; pN, pathological regional lymph node; pM, pathological distant metastasis; pStage, pathological staging. FPG, fasting plasma glucose.

Case No.	Age	Sex	Size (mm)	WHO2017	Ki67 (%)	pT	pN	pM	pStage	HbA1c (%)	FPG (mg/dL)	Insulin (mIU/L)
**1**	81	Male	14	G1	1	1	0	0	I	4.6	76	19.5
**2**	38	Female	14	G1	1	1	0	0	I	4.5	50	18.3
**3**	14	Male	25	G1	1	2	0	0	II	4.4	54	32.8
**4**	68	Female	20	G1	1	2	1	0	II	5.3	63	7.0
**5**	44	Female	12	G1	1	2	1	0	II	4.1	61	12.6
**6**	77	Female	18	G1	1	1	0	0	I	5	4.6	26.3
**7**	53	Female	14	G1	2	1	0	0	I	5	29	32.2
**8**	79	Female	10	G1	2	1	0	0	I	4.5	65	4.0
**9**	78	Male	15	G1	2	1	0	0	I	5.1	56	23.7
**10**	60	Female	13	G2	3	1	1	0	III	4.3	56	17.7

Supplementary table 2

The staging was based on the AJCC 8th edition. pT, pathological primary tumor; pN, pathological regional lymph node; pM, pathological distant metastasis; pStage, pathological staging

Case no.	Age (years)	Sex	Max Size (mm)	WHO2017	Ki67 (%)	pT	pN	pM	pStage	HbA1c (%)
**1**	67	Male	15	G1	< 1	1	0	0	Ⅰ	6.3
**2**	78	Male	12	G1	< 1	1	0	0	Ⅰ	6.9
**3**	66	Male	9	G1	< 1	1	0	0	Ⅰ	5.5
**4**	54	Female	10	G1	< 1	1	0	0	Ⅰ	5.9
**5**	72	Male	9	G1	< 1	1	0	0	Ⅰ	6.8
**6**	72	Male	4	G1	1	1	0	0	Ⅰ	6.5
**7**	58	Male	9	G1	1	1	0	0	Ⅰ	7.9
**8**	50	Male	15	G1	1	1	0	0	Ⅰ	5.7
**9**	54	Male	20	G1	1	2	0	0	Ⅱ	6.4
**10**	43	Male	18	G1	1	1	0	0	Ⅰ	5.6
**11**	58	Male	8	G1	1	1	0	0	Ⅰ	6.3
**12**	62	Female	15	G1	2	1	0	0	Ⅰ	7.7
**13**	59	Male	12	G1	2	1	0	0	Ⅰ	6.8
**14**	67	Male	8	G1	2	1	0	0	Ⅰ	6.3
**15**	67	Female	10	G1	2	1	0	0	Ⅰ	5.8
**16**	71	Male	10	G1	2	1	0	0	Ⅰ	6.5
**17**	45	Female	22	G1	2	2	0	0	Ⅱ	5.7
**18**	48	Male	9	G1	2	1	0	0	Ⅰ	7.2
**19**	54	Female	45	G1	2	3	0	1	Ⅳ	6.5
**20**	72	Male	8	G1	2	1	0	0	Ⅰ	6
**21**	68	Male	17	G1	2	2	0	0	Ⅱ	6.7
**22**	47	Male	30	G2	4	2	0	0	Ⅱ	5.5
**23**	54	Male	7	G2	5	1	0	0	Ⅰ	6
**24**	42	Female	30	G2	5	2	0	0	Ⅱ	5.4
**25**	49	Male	27	G2	5	2	0	0	Ⅱ	5.8
**26**	60	Female	12	G2	5	1	0	1	Ⅰ	7.1
**27**	47	Female	22	G2	5	2	0	0	Ⅱ	5.6
**28**	71	Female	21	G2	5	2	0	0	Ⅱ	5.8
**29**	62	Male	52	G2	10	3	1	1	Ⅳ	7.4
**30**	38	Female	40	G2	15	3	1	1	Ⅳ	7.6
**31**	66	Female	65	G3	50	3	0	1	Ⅳ	7.1
